# Multimodal Management of Febrile Infection-Related Epilepsy Syndrome in a 17-Year-Old Male

**DOI:** 10.7759/cureus.44412

**Published:** 2023-08-30

**Authors:** Erin E Bellingham, Caroline G Hammond, Hanna S Sahhar, Sami E Rishmawi

**Affiliations:** 1 Pediatrics Department, Edward Via College of Osteopathic Medicine - Louisiana Campus, Monroe, USA; 2 Pediatrics Department, Edward Via College of Osteopathic Medicine - Carolinas Campus, Spartanburg, USA; 3 Pediatric Intensive Care Unit, Spartanburg Regional Healthcare System, Spartanburg, USA

**Keywords:** fires, seizure medications, refractory status epilepticus, pediatric seizure, norse

## Abstract

New-onset refractory status epilepticus (NORSE) is a clinical presentation, not a specific diagnosis, in which healthy people are suddenly struck by prolonged seizures that do not respond to at least two anti-seizure drugs and do not have a clear structural, toxic, or metabolic cause.Febrile infection-related epilepsy syndrome (FIRES) is considered a sub-category of NORSE. Our patient is a 17-year-old male admitted to the pediatric ward after a self-limited convulsive episode at home, noted to occur following five days of upper respiratory infection symptoms accompanied by fever. After multiple generalized tonic-clonic seizures necessitating treatment, he went into status epilepticus despite multiple antiepileptic drugs. The possibility of FIRES had been considered from the onset of refractory status epilepticus; as a result, an intensive multimodal treatment regimen was proactively implemented with some clinical improvement.

## Introduction

The designation of new-onset refractory status epilepticus (NORSE) is given to patients when there is no history of epilepsy or evidence of an underlying structural, metabolic, or toxic precipitant for intractable seizures. An etiology is identified in about half of NORSE cases in adults, with the majority attributed to auto-immune encephalitis. However, many cases of NORSE remain cryptogenic [1].

Pediatric cases of acute-onset intractable seizures accompanied by encephalopathy were first reported in the 1960s [2]. The syndrome was described under multiple names, including devastating epileptic encephalopathy in school-aged children (DESC) [3], “idiopathic catastrophic epileptic encephalopathy” [4], “severe refractory status epilepticus due to presumed encephalitis” [5], or acute encephalitis with refractory repetitive partial seizures (AERRPS) [6] before eventually receiving a new name: febrile infection-related epilepsy syndrome (FIRES) [7]. FIRES is considered a subcategory of NORSE with additional clinical features [8].

FIRES is typically diagnosed when, in addition to meeting the criteria for NORSE, a pediatric patient demonstrates treatment-resistant status epilepticus following a febrile illness, and the onset of seizures is accompanied by neuropsychological impairment [8]. FIRES occurs most frequently in children with a median age of six to eight years and is more common in boys than girls [1,8]. The syndrome must be preceded by a febrile illness beginning two days to two weeks prior to NORSE. This prodromal phase typically features a nonspecific upper respiratory or gastrointestinal illness. New-onset seizure activity abruptly follows, and patients demonstrate status epilepticus refractory to multiple anti-epileptic drugs (AEDs). Most survivors have varying degrees of long-term neurologic sequelae, including cognitive impairment and functional disability [1]. There are worse outcomes associated with younger patient age at onset, use of general anesthesia, administration of multiple anesthetic agents, and iatrogenic complications incurred during treatment. There may be worse outcomes associated with increased duration of burst suppression coma, but it is unclear whether this is due to the intervention itself or the severity of the disease at diagnosis [8-9].

The seizures may be either generalized or multifocal in nature with varying levels of awareness and subsequently evolve into a refractory status epilepticus in which patients may endure hundreds of seizures daily [8]. Electroencephalogram (EEG) typically shows global background slowing with multifocal seizure activity predominantly in the frontal and temporal lobes. Typical EEG characteristics of FIRES include initial seizures that are short and sparse with a progressive deterioration to status epilepticus, beta-delta complexes that can resemble extreme delta brush, seizures beginning with a prolonged focal activity that evolves to rhythmic spikes or spike-wave complexes, and shifting ictal activity [10].

FIRES is a diagnosis of exclusion and is distinct from other diagnoses, such as autoimmune encephalitis (AIE), which presents with positive neuroimaging and antibody testing [11]. The initial brain MRI in patients with FIRES is usually unremarkable. In the chronic phase of FIRES, MRI changes, such as hippocampal sclerosis and focal and diffuse cortical atrophy, become evident in some patients [12]. Such changes are associated with neurologic and psychiatric impairment [8].

Due to the rarity of the syndrome, there is little evidence to guide the treatment of FIRES. Therefore, the goal of treatment in the acute phase is to control status epilepticus. Patients typically require multiple AEDs to suppress seizures; successful combinations of first- and second-line medications vary among FIRES patients [2]. It is not uncommon for patients to require multiple intravenous anticonvulsant infusions in addition to four to six AEDs to attain seizure control in the acute phase. An effective therapeutic option is pharmacologic burst suppression coma with intravenous barbiturate, benzodiazepine, and ketamine infusion, but prolonged burst suppression may be associated with longer intensive care unit (ICU) stay and worse cognitive outcomes. In addition, first-line management of FIRES requires immune modulation with interventions such as steroids, intravenous immune globulin (IVIG), plasma exchange, and ketogenic diet [2, 13]. Second-line immune modulation with an interleukin-1 receptor antagonist such as anakinra can be pursued if there is no improvement with AEDs and first-line immunomodulatory therapy [2].

FIRES is associated with a mortality rate of 10-12%, most often secondary to complications, such as respiratory failure, sepsis, cardiac arrest, intracranial hemorrhage, hypotension, or multiorgan failure [2]. The median duration of ICU stay is 20-40 days regardless of outcome [1]. Survivors of the acute phase experience considerable morbidity in the chronic phase. Very few children with FIRES attain seizure freedom, and most remain on multiple AEDs. Younger age is associated with a more severe course of disease and poorer cognitive outcomes. However, regardless of age, neurocognitive deficits and behavioral problems are exceedingly common in patients who survive the acute phase of FIRES. Deficits in speech, attention, and executive function have been documented with varying degrees of severity [2]. Due to the long-term morbidity of FIRES, optimization of treatment is of utmost importance.

## Case presentation

A 17-year-old male presented to the urgent care with his mother complaining of headache, arthralgia, myalgia, congestion, and sore throat for three days. Vital signs were within normal limits, except for a high fever of 39.4 °C. Cepheid reverse transcriptase polymerase chain reaction (RT-PCR) testing for influenza A and B, respiratory syncytial virus (RSV), and severe acute respiratory syndrome coronavirus 2 (SARS-CoV-2) was negative, and the patient was sent home for supportive care, presuming a viral upper respiratory infection. 

On the fifth day of illness, the patient was brought to the Emergency Department (ED) via emergency medical services (EMS) transport following a self-limited convulsive episode at home, characterized by generalized “shaking.” The patient was unresponsive to external stimuli during the episode, which lasted an estimated 1-2 minutes. No bladder or bowel incontinence was reported. He had no personal or family history of seizures. He was somnolent and minimally interactive for the duration of the ED encounter; lorazepam was given. The remaining physical examination was noncontributory, demonstrating no head trauma, meningeal signs, focal neurologic deficits, respiratory distress, cardiovascular abnormalities, rash, or mucosal lesions. Computed tomography of the head was unremarkable. Broad spectrum antibiotic was administered intravenously. Laboratory values were notable for mild thrombocytosis with platelet count elevated at 354,000 per microliter (RR 119-332 * 103 platelets/uL), but the remainder of the blood count was within normal limits. Urinalysis was unremarkable. Urine drug screen was positive for cannabinoids and benzodiazepines, the latter having been administered in the ED. The patient was transferred for inpatient pediatric care. 

Upon admission, lumbar puncture was deferred because the patient lacked fever or meningeal signs, initial labs were largely unremarkable, and antibiotics had already been given in the ED. The morning after admission, the patient was febrile at 38.9 °C and experienced two successive generalized tonic-clonic seizures within 30 minutes, the second of which was captured on EEG and terminated with the administration of lorazepam. EEG demonstrated a 90-s generalized seizure, characterized by rhythmic high-amplitude theta-delta frequencies bifrontally that evolved into polyspike and wave discharges at a frequency of 3 Hertz, followed by diffuse postictal suppression. The patient was moved to the pediatric ICU in which fosphenytoin bolus and maintenance plus levetiracetam at maintenance dosing were started. MRI with and without contrast was unremarkable.

**Figure 1:**
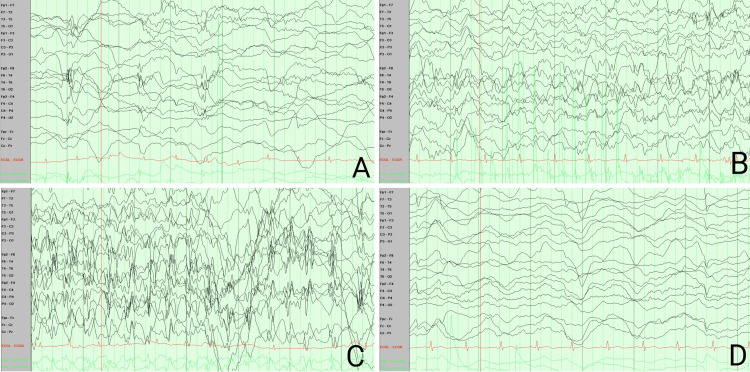
Diffusely slowed background rhythms preceding a single electrographic seizure on hospital day three, which demonstrated bilateral propagation from a right frontotemporal focus.

Lumbar puncture resulted in a traumatic tap, and cerebrospinal fluid (CSF) analysis demonstrated 7,161 erythrocytes and 31 leukocytes with a 63% predominance of segmented neutrophils. Corrected leukocyte count was consistent with moderate pleocytosis of 31 white blood cells per microliter (RR 0-5 * 106 cells/uL). CSF Gram stain was negative, CSF glucose was 74 mg/dL (RR 50-80 mg/dL), and CSF protein was 57 mg/dL (RR 14-45 mg/dL). The BioFire FilmArray Meningitis/Encephalitis (ME) Panel, which included Streptococcus pneumoniae, Haemophilus influenzae, Neisseria meningitidis, Listeria monocytogenes, herpesviruses, enterovirus, parechovirus, and cryptococcus, among others, was unanimously negative. A CSF sample was sent for comprehensive antibody evaluation for autoimmune and paraneoplastic encephalitis. On hospital day two, despite fosphenytoin and levetiracetam, repetitive breakthrough seizure activity persisted, necessitating IV lorazepam. The patient was placed on IV midazolam infusion; methylprednisolone and valproate were added to the regimen. EEG demonstrated diffusely slowed background rhythms that were largely synchronous and symmetric, consisting of low-amplitude theta waves and moderate-amplitude delta waves at 1-2 Hertz in the frontal lobes, compatible with encephalopathy. A single electrographic seizure originated from the right frontotemporal region and subsequently generalized with polyspikes and slow waves bilaterally before postictal suppression (Figure 1, A-D).

Personality changes and violent behavior began to dominate the clinical picture with no return to the neurocognitive baseline. Comprehensive antibody testing for autoimmune disease, which included antinuclear and double-stranded DNA antibodies, rheumatoid factor, thyroid peroxidase antibodies, and Sjogren’s antibodies, among others, was pursued; all results were unrevealing. Repeated laboratory values were remarkable only for a C-reactive protein (CRP) level of 5.9 mg/dL (NR 0.0-0.6 mg/dL). A ketogenic diet was initiated and a trial of midazolam taper, but his agitation and violent behavior escalated despite adding dexmedetomidine infusion and haloperidol as a PRN medication. Escalation of violent behavior prompted the initiation of propofol infusion. The patient was subsequently intubated due to respiratory depression.

Lumbar puncture was repeated. CSF analysis demonstrated a leukocyte count of 11, a decrease from the prior 31 white blood cells noted on the initial traumatic tap (NR 0-5 * 106 cells/uL). Otherwise, Gram stain and cultures were negative; CSF glucose and protein were within normal limits. Repeat BioFire FilmArray Meningitis/Encephalitis (ME) panel was again negative. Laboratory values were significant for a CRP that had risen to 11.1 mg/dL (NR 0.0-0.6 mg/dL), serum phenytoin of 23.1 ug/mL (therapeutic range 10.0-20.0 ug/mL), and valproate that was therapeutic at 55 mcg/mL (therapeutic range 50.0-100.0 mcg/mL). Electrolytes were within normal limits. The second MRI of the brain with contrast arteriography and venography (MRA and MRV) was unremarkable, demonstrating no abnormal signal hyperintensities or areas of diffusion restriction.

Subsequent EEG showed diffuse background slowing with moderate amplitude bifrontal delta activity at 1-2 Hz. Intermittent central beta activity at 15 Hz was predominantly symmetric and synchronous (Video 1).

**Video 1:** Super-refractory status epilepticus (SRSE) was diagnosed when seizure activity recurred with a taper and discontinuation of the anesthetic regimen. The initially focal seizure subsequently generalized.

Lacosamide was added to the existing valproate and levetiracetam regimen. High-dose pyridoxine was added to the ketogenic formula once daily for five days. A course of intravenous immune globulin modulation was pursued with two days of therapy, reaching a cumulative dose of 2 g/kg, and topiramate was added to the antiepileptic regimen. Repeated MRI brain for the third time remained unremarkable, and attentiveness to temporal lobes gave no indication of mesial temporal sclerosis. Quetiapine was added due to severe episodes of agitation.

Despite these measurements, the patient continued to experience focal seizures with impaired awareness and oxygen desaturation. Between seizures, he was occasionally alert and attentive to care activities; however, his affect remained labile. He experienced an episode of behavioral arrest with staring and desaturation suggestive of absence seizure and an episode of paroxysmal autonomic instability with bradycardia and oxygen desaturation. Subsequent EEG demonstrated background waking rhythms with improved organization and moderate voltage; occipital leads showed symmetric and poorly reactive 9-10 Hertz alpha rhythm. However, no epileptiform activity was captured to confirm the seizure manifestations that were observed clinically. Anakinra was added, and valproate was supplanted by clobazam due to persistent transaminase elevation as well as alkaline phosphatase and gamma-glutamyl transferase. Following these changes, the patient continued to experience focal and absence seizures with autonomic instability approximately once daily, but the episodes were self-limited (Video 2). Swallowing evaluations were subsequently initiated, with improvement eventually permitting normal oral intake before discharge.

**Video 2:** The patient experienced a focal seizure with autonomic instability and oxygen desaturation on hospital day 24. When oxygen desaturation persisted, lorazepam was administered, and seizure activity ceased.

As seizure frequency decreased, midazolam and dexmedetomidine were weaned entirely. His mental status remained below baseline; he recognized and asked for immediate family members but did not recognize members of the care team or cooperate with treatment, refusing both EEG and ketogenic meals. When asked about school or other past activities, the patient grew agitated and tremulous, demonstrating perseveration of single words and movements. He had little recollection of his day-to-day activities prior to admission. Although he remained oriented to himself, he was frequently disoriented by place, time, and situation.

The antibody evaluation for autoimmune and paraneoplastic encephalitis, which included N-methyl-D-aspartate receptor (NMDAR) antibodies, anti-α-amino-3-hydroxy-5-methyl-4- isoxazolepropionic acid receptor antibodies, gamma-aminobutyric acid A and B receptor antibodies, neuromyelitis optica aquaporin-4 antibodies, myelin oligodendrocyte glycoprotein antibodies, and more than 20 other causative antibodies, was also entirely negative. The patient’s family declined neurological rehabilitation, so he was discharged home on day 29. At outpatient follow-up one week later, he demonstrated mild persistent disorientation with no other neurocognitive deficits. His guardian reported no further seizures on the AED regimen consisting of clobazam, lacosamide, levetiracetam, and topiramate.

## Discussion

We discuss a 17-year-old male patient who received the diagnosis of FIRES, an exceedingly rare diagnosis of exclusion characterized by NORSE several days after a common febrile illness [2,14]. His clinical course, consisting of a flu-like syndrome followed by intractable seizures, is suggestive of a nonmicrobiological underpinning, corroborated by thoroughly negative microbiological analyses. The patient’s isolated mild pleocytosis was in keeping with CSF studies in other documented FIRES patients, which demonstrated mild-to-moderate pleocytosis in about half of cases in one series [7,14]. While other precipitants and diagnoses were considered, the patient’s characteristic timeline of febrile illness and subsequent NORSE with negative laboratory studies and neuroimaging was suggestive of FIRES, prompting increased vigilance for refractoriness and expedient initiation of multimodal therapy.

To further complicate the workup of refractory seizures following a febrile syndrome of no discernible microbial or immune etiology, the patient’s initial urine drug screen was positive for tetrahydrocannabinol (THC). Parental reports of marijuana use suggested the possibility of synthetic cannabinoid (SC) contamination as the precipitant of seizure activity accompanied by agitation and violent behavior. However, status epilepticus secondary to synthetic cannabinoid toxicity has demonstrated dose dependency in most cases. It is rare and generally self-limited, and the patient’s guardian described his THC use as intermittent in frequency [15]. Additionally, the onset of behavioral symptoms with excessive SC use is typically expeditious, whereas alteration in mental status in this patient did not become apparent until hospital day four [15].

Postictal psychosis (PIP) following the onset of seizures was also considered a differential diagnosis. However, the development of PIP typically follows the onset of a seizure disorder by an interval of several years [16].

Additionally, the patient’s disorientation and violent behavior demonstrated a fluctuating course that worsened overnight, more typical of delirium. When the patient was oriented, delusional thought content was not observed [17]. Responsiveness to internal stimuli was not apparent at any point in the clinical course. Interestingly, other patients ultimately diagnosed with FIRES have characteristically demonstrated severe interictal confusion during the acute phase followed by neurocognitive decline, behavioral problems, and learning disabilities [7-8,18]. The severe memory deficits and persistently altered mental status evident in the patient here described could be entirely attributable to FIRES.

The patient demonstrated predominantly focal seizures, with occasional propagation to bilateral hemispheres. One such seizure with a right frontotemporal focus that subsequently spread bilaterally was captured electrographically on the second hospital day. However, the patient was often agitated and unable to cooperate with long-term EEG when not heavily sedated; he was apt to remove the electrodes himself. Many episodes were therefore observed but not captured, including focal, absence, and autonomic seizures. Absence and autonomic seizures developed later in the hospital course, and both behavioral arrest and autonomic instability responded to intravenous benzodiazepine administration.

Interictal EEG monitoring was nonspecific. The patient demonstrated diffusely slowed delta-theta background activity suggestive of encephalopathy [19]. It was consistent rather than intermittent and improved by the end of the hospital course when the patient demonstrated moderate organization and increased but poorly reactive alpha rhythms. No triphasic waves were observed [19]. There was no evidence of the extreme delta brush seen in some cases of NMDAR antibody-positive AIE, and no specific electrographic findings have been found to typify other AIE subtypes [20-21]. Other patients ultimately diagnosed with FIRES have also demonstrated slowed background activity and focal discharges unaccompanied by causative metabolic disturbances [18,22]. However, due to a lack of specificity, EEG findings in FIRES remain supportive rather than diagnostic.

The patient’s persistently unremarkable MRI was consistent with the exclusionary diagnosis itself and with MRI characteristics of other FIRES patients. Structural abnormalities on initial neuroimaging rule out FIRES as a probable cause of refractory status epilepticus [2]. In fact, many patients ultimately diagnosed with FIRES have entirely unremarkable initial imaging studies [7,14,23]. In those with positive MRI, the most common findings are temporal and hippocampal signal hyperintensities or edema with no structural lesions [14,23]. However, significant structural changes frequently occur in the chronic phase of FIRES, including diffuse cortical atrophy in over half of patients with MRI follow-up in one study [14]. In those without diffuse atrophy, sclerosis of the hippocampi was evident in several patients. Murine studies have suggested that diffuse cortical atrophy would likely result from intractable status epilepticus of any etiology, but in any case, it is associated with poor outcomes overall [2]. Most survivors demonstrate some degree of neurocognitive disability that remains stable despite rehabilitation measures, and some remain in a persistent vegetative state [2,22]. The patient described here demonstrated notable memory impairment, speech difficulty, episodic verbal and motor perseveration, poor coordination, and behavioral problems. Although their frequency had decreased, his seizures posed an ongoing risk for falls. Over 90% of patients who survive the acute phase of FIRES experience lifelong seizures refractory to antiepileptic drug therapy. These tend to be stable in the long term, neither progressively worsening nor improving [2]. The patient in this case demonstrated approximately two self-limited focal seizures daily near the end of the inpatient treatment period, but imaging continued to be unrevealing throughout the hospital course. Persistently unremarkable MRI while inpatient should theoretically be a positive prognostic factor, but the correlation of imaging characteristics and clinical outcome is historically lacking during the acute phase of FIRES [2,14].

Due to its frequently catastrophic onset, patients ultimately diagnosed with FIRES often receive therapeutic regimens so multimodal that clinical improvements are difficult to trace to single interventions; such was the case with the patient here described. Initial status epilepticus protocol consisting of fosphenytoin and levetiracetam was accompanied by intravenous methylprednisolone and midazolam; this regimen resulted in cessation of electrographic seizure activity after the third hospital day, a treatment response uncharacteristic of FIRES. However, his severely altered mental status persisted. His violent behavior required intensive sedation measures and eventually intubation. Due to seizure activity upon withdrawal of propofol, the patient was categorized as super-refractory and treated with midazolam burst suppression plus intravenous immune globulin [24]. The treatment period was capped at 72 hours given statistically worse neurocognitive prognoses in FIRES patients treated with prolonged burst suppression, but more research is needed to analyze cognitive outcomes according to pharmacologic agent, severity of patient presentation, and treatment duration [8,18]. As is often the case in FIRES, the patient demonstrated persistent and evolving seizure activity after burst suppression. However, because control of status epilepticus in the acute phase is crucial to reduce neurologic sequelae, a burst suppression period of limited duration is both reasonable and beneficial if multiple AEDs are insufficient [2].

Following pharmacologic burst suppression, the medication regimen was changed to mitigate iatrogenic complications while targeting the hypothesized inflammatory underpinning of FIRES. Anakinra, a recombinant IL-1 receptor antagonist, has demonstrated some degree of benefit in FIRES by decreasing the length of hospital stay in one retrospective study [8,25]. Though not statistically significant, earlier initiation of anakinra was associated with decreased seizure frequency in some patients as well. In the cohort studied, hepatic enzyme elevation was noted in only one patient of 25 [25]. However, this was clearly a consideration in the patient described here, whose hepatic enzymes and ammonia levels increased following two additions to his AED regimen during the burst suppression period, but did not meet clinical chemistry criteria for drug-induced liver injury (DILI) at any point in the hospital course [26]. However, his laboratory results were sufficient to prompt the substitution of valproate for clobazam when anakinra was introduced for a 12th-day course. Following the change in his AED regimen, the patient’s laboratory values steadily improved. He also demonstrated a continued decline in seizure frequency.

The patient was unwilling to cooperate with a ketogenic diet, one of the few interventions with documented success in FIRES, and his cognitive status prohibited explanation of therapeutic efficacy as a means of encouraging compliance. However, no increase in seizure frequency was noted with abrupt cessation of ketosis for the last two days prior to discharge. While the ketogenic diet has successfully controlled seizures in several patients with FIRES and other treatment-resistant epilepsies, results have not been reproducible in all investigations, suggesting that while the ketogenic diet yields amazing results in some patients, it is not efficacious for seizure control in all cases [2,8,14,18]. Ultimately, there is much research to be done with regard to treatment optimization and pathogenesis, and elucidation of the latter will likely expedite the former.

## Conclusions

The 17-year-old patient described in this case presented with NORSE several days following the onset of a nonspecific febrile illness and was ultimately diagnosed with FIRES, a rare epileptic encephalopathy without a structural, infectious, toxic, or autoimmune underpinning. The patient’s seizures were controlled with proactive escalation of the status epilepticus treatment protocol. Chronic epilepsy and neurocognitive impairment are common in patients with FIRES, and mitigation of such sequelae is the motive for further study of its pathogenesis and advancement of management strategies.
